# Thermo-Mechanical Fatigue Behavior and Resultant Microstructure Evolution in Al-Si 319 and 356 Cast Alloys

**DOI:** 10.3390/ma16020829

**Published:** 2023-01-15

**Authors:** Kun Liu, Shuai Wang, Lei Pan, X.-Grant Chen

**Affiliations:** 1 Department of Applied Science, University of Quebec at Chicoutimi, Saguenay, QC G7H 2B1, Canada; 2 Arvida Research and Development Centre, Rio Tinto Aluminum, Saguenay, QC G7S 4K8, Canada

**Keywords:** Al-Si casting alloys, thermo-mechanical fatigue, precipitates, fatigue life prediction, fracture

## Abstract

The out-of-phase thermo-mechanical fatigue (TMF) behavior of the two Al-Si cast alloys most widely used for engine applications (319 and 356) were investigated under temperature cycling (60–300 °C) and various strain amplitudes (0.1–0.6%). The relationship between the microstructural evolution and TMF behavior was closely studied. Both alloys exhibited asymmetric hysteresis loops with a higher portion in the tensile mode during TMF cycling. The two alloys showed cyclic softening behavior with regard to the maximum stress, but an earlier inflection of cyclic stress was found in the 356 alloy. The TMF lifetime of the 319 alloy was generally longer than that of the 356 alloy, especially at higher strain amplitudes. All the precipitates (β′-MgSi in 356 and θ′-Al_2_Cu in 319) coarsened during the TMF tests; however, the coarsening rate per cycle in the 356 alloy was significantly higher than that in the 319 alloy. An energy-based model was applied to predict the fatigue lifetime, which corresponded well with the experimental data. However, the parameters in the model varied with the alloys, and the 356 alloy exhibited a lower fatigue damage capacity and a higher fatigue damage exponent.

## 1. Introduction

Al-Si cast alloys are widely used in modern automotive industries to replace cast iron-fabricated engine components (engine blocks and cylinder heads) due to their high strength-to-weight ratio, excellent castability, and thermal conductivity [1,2,3,4]. In combustion engine applications, components undergo complex loading changes and dramatic temperature gradients during start-up and shutdown cycles. For example, engines are often heated from ambient temperature—or even lower in cold winters (−25 °C)—to 250–300 °C and, vice versa, rapidly cooled down in start–stop cycles [5]. Due to the large thermal gradient, significant thermal and mechanical stresses occur in the engine components because of the changing thermal expansion/contraction of various engine components [6]. As a result, cyclic stress and temperature changes can result in thermo-mechanical fatigue (TMF) [4,6], which can lead to serious failures, significantly limiting components’ service life. Therefore, understanding TMF behavior is increasingly important for the design, reliability assessment, and lifecycle management of critical engine components during their industrial application. It has been reported [4,5,6] that the most significant damage mechanism in engine components is out-of-phase (OP) TMF cycling, where the maximum mechanical strain occurs at the minimum temperature.

Several studies have evaluated the TMF behavior in Al-Si cast alloys [7,8,9,10,11,12,13]. Huter et al. [11] performed TMF tests on Al-Si-Cu and Al-Si-Mg cast alloys with varying Si, Cu, Fe, and Sr contents and reported that the nucleation and propagation of cracks were predominantly influenced by the eutectic Si and Fe-rich intermetallics, which lowered the fatigue resistance. Sehitoglu et al. [14] reported similar results for the Al-Si 319 alloy, finding that the alloy presented worse TMF resistance at higher Fe contents due to a lower stress level and higher softening speed. It was also found that the rapidly solidified material with a small secondary dendrite arm space (SDAS) presented the highest cyclic stress–strain amplitude, whereas the stress levels decreased gradually as the SDAS levels increased [14]. It has also been reported that a finer SDAS, lower porosity level, and lower brittle intermetallic content are beneficial for better thermal resistance and, hence, the fatigue life of defect-free A356 alloys can be improved by at least one order of magnitude [15,16].

A recent study employed ultrasonic melt treatment for Al-Si cast alloys to refine the grain size, the eutectic Si, and the Fe-rich intermetallics, which significantly enhanced the fatigue life [17]. However, contradictory results regarding the influence of precipitates on the TMF resistance of Al-Si cast alloys can be found in the literature. For example, Azadi et al. [8,9] studied the effect of heat treatment and strain rate on the TMF cyclic behavior of the A356 alloy and found that the TMF life was not significantly affected by the heat treatment in the as-cast and T6 conditions. In contrast, Moizumi et al. [18] reported that the thermal fatigue life of an A356 + 1%Cu alloy in the over-aged condition was doubled compared to the peak-aged condition. Hunter et al. [11] also reported that the TMF resistance of an Al-Si-Cu cast alloy was improved compared to that of a Cu-free Al-Si alloy due to the Cu-containing precipitates. Beck et al. [19] studied the influence of high-cycle fatigue (HCF) loading on the TMF life of Al-Si-Mg/Cu cast alloys in the T6 condition and concluded that TMF was not significantly affected by HCF loading at low strain amplitudes and that precipitation in the T6 condition was effected by HCF. Toda et al. [13] reported that precipitates preferentially oriented perpendicular to the loading direction can effectively prolong the in-phase TMF life, but the study did not comment on the OP-TMF life. Toyoda et al. [20] investigated the microstructural change in an Al-Si-Cu-Mg cast alloy under thermo-mechanical loading conditions and reported that the precipitates were observed to align perpendicularly to the compressive stress during heating, but they did not further discuss how these precipitates with preferential orientations affected the TMF behavior. Therefore, the systematic evolution of precipitates during TMF under various strain amplitudes, which is one of the significant factors influencing the TMF resistance, is still not well-documented in Al-Si cast alloys.

Contemporary engine components are designed to increase the maximum operating temperature and pressure in order to further reduce greenhouse gas emissions and improve their fuel efficiency [5,19,21,22]. However, the TMF behaviors reported in the literature are limited to relatively low maximum temperatures of 200 or 250 °C [9,11,16,18,19], and the TMF behavior at higher maximum temperatures (approximately 300–350 °C) is seldom reported. Moreover, the microstructural evolution during TMF over a wider temperature range, especially precipitation in age-hardening aluminum alloys, is still not fully understood. This study selected two of the most widely used Al-Si cast alloys—namely, Al-Si 356 and Al-Si-Cu 319—to perform OP-TMF under temperature cycling (60–300 °C) and various strain amplitudes (0.1–0.6%). The TMF behaviors were evaluated using the strain and stress cyclic response, softening rate, and fatigue life assessment. The evolution of the precipitates during TMF cycling was characterized using transmission electron microscopy (TEM) to establish the relationship between the microstructure and TMF behavior. Additionally, the TMF damage mechanism is discussed and an energy-based model for the prediction of the TMF lifetimes of the two alloys is introduced.

## 2. Materials and Methods

Two Al-Si cast alloys (319 and 356) were prepared using commercially pure aluminum (99.7%), pure Mg (99.9%), and Al-50%Si, Al-25%Mn, Al-50%Cu, Al-10%Sr, and Al-5%Ti-1%B master alloys. The raw materials were batched in clay-graphite crucibles and melted using an electric resistance furnace. After reaching 780 °C, the melt was held for 30 min and then degassed with high purity argon gas for 15 min. The melt was then cast into a permanent wedge mold pre-heated at 250 °C. The chemical compositions of the two alloys were analyzed using optical emission spectroscopy, and the results are listed in Table 1. All the as-cast samples were subjected to a two-step solution treatment followed by water quenching. Artificial aging was then performed at 200 °C for 5 h to achieve the T7 temper condition. Table 2 summarizes the parameters of the heat treatment applied to the alloy samples.

**Figure 1:**
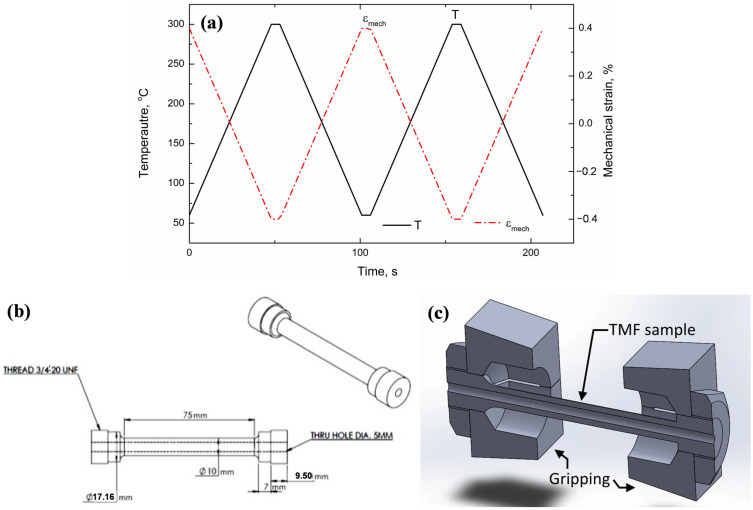
Schematic illustration of OP-TMF at 0.4% strain amplitude (**a**), TMF sample dimensions (**b**), and the setup with gripping blocks (**c**).

After the T7 heat treatment, the TMF specimens were machined to a parallel gauge length of 75 mm and diameter of 10 mm and a 5 mm diameter hole was machined through the specimens for air circulation, as schematically shown in Figure 1. In the present work, OP-TMF, where the highest mechanical strain is reached at the lowest temperature, was applied to the experimental alloys (as shown in Figure 1a). OP-TMF tests were performed using a Gleeble 3800 thermal–mechanical simulator system with a strain-controlled loading mode. The samples were heated using a Joule heating system and cooling was achieved by using compressed air delivered through a hollow tube specimen. The TMF temperature was varied between 60 and 300 °C for a cycle using heating and cooling rates of 5 °C/s. The temperature was measured and controlled using thermocouples attached to the center of the gauge length. The applied mechanical strain amplitudes were 0.1, 0.2, 0.4, and 0.6%. The TMF tests were automatically terminated when either a total of 2000 fatigue cycles was reached or a decrease of 30% in the initial maximum tensile/compression stress was detected. When the TMF test was terminated without full fracture of the sample, the sample was pulled to full fracture in the Gleeble machine at room temperature for a later fracture analysis. More details on the TMF test procedure, such as the process to reach zero stress at the beginning of the test and the isolation of the mechanical strain from the total strain during the TMF test, can be found in our previous study [23].

The microstructures of the TMF samples were characterized using optical and electron microscopies. Optical microscopy was used to observe the as-cast and T7 grain structures and intermetallics, whereas a scanning electron microscope (SEM) equipped with an energy-dispersive X-ray spectroscope was used to identify the intermetallic phases and to observe the fracture faces after the TMF tests. TEM was used to observe the evolution of the precipitates during TMF testing. TEM samples were cut from the cast ingot after the T7 treatment and from the gauge zone of the samples (close to fracture) after the TMF tests at various strain amplitudes. The TEM samples were initially punched into 3 mm diameter disks and then ground and polished to approximately 30 μm. The samples were then twin-jet electrochemically polished using a solution of 75 mL HNO3 in 250 mL methanol at −30 °C to create TEM observation regions. Image analysis was used to quantify the characteristics of the intermetallics and precipitates, such as their area fraction and number density, under various conditions.

## 3. Results

### 3.1. TMF Behaviors

**Figure 2:**
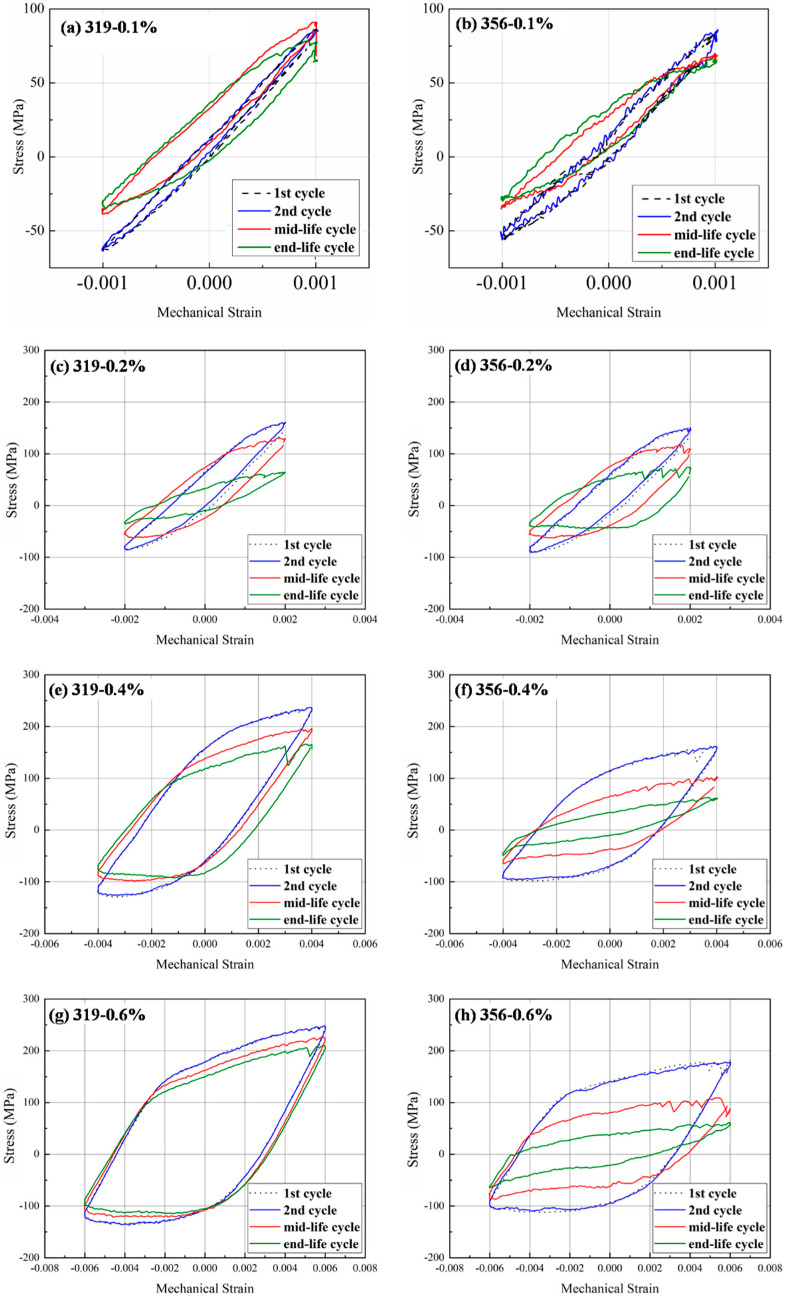
OP-TMF stress–strain hysteresis loops for experimental alloys at different strain amplitudes of 0.1, 0.2%, 0.4%, and 0.6%: (**a**,**c**,**e**,**g**) 319 alloy; (**b**,**d**,**f**,**h**) 356 alloy.

Figure 2 shows the stress–strain hysteresis loops of the initial, mid-life, and end-life cycles of the two alloys at the mechanical strain amplitudes of 0.1, 0.2, 0.4, and 0.6%. Both alloys exhibited some common phenomena. First, both alloys exhibited asymmetrical hysteresis loops at all strain amplitudes, and the tensile portions were higher than the compression portions, indicating a higher tensile stress than compression stress at the same tensile/compression strain, which has also been reported in the literature [8,11,14,18,23]. This can be attributed to the characteristics of OP-TMF, where the maximum tensile strain is reached at the lowest temperature (60 °C) and the compression strain increases to a maximum at the highest temperature (300 °C). The mechanical properties of Al-Si cast alloys worsen with increasing temperature [5,24,25]. Therefore, the stress needed to reach the desired strain was higher in the tensile mode due to its low temperature compared to the compression mode at high temperature, leading to asymmetrical hysteresis loops with higher portions in the tensile mode (stress > 0 MPa in Figure 2). Second, the loop areas were significantly larger and the peak tensile/compression stress increased with increasing strain amplitude. As shown in Figure 2a,c,e,g, the loop area of the 319 alloy gradually increased with an increase in the strain amplitude from 0.1 to 0.6%, whereas the mid-life peak tensile stress increased from 100 MPa at 0.1% to 124, 198, and 211 MPa at 0.2, 0.4, and 0.6%, respectively. A comparison of the mid- and end-life cycles with the first cycle showed that the shape of the hysteresis loop became flatter with increasing fatigue life at a fixed strain amplitude, indicating an increasing plastic strain, which can be calculated using the strain range at zero stress. Additionally, the maximum tensile and compression stresses decreased at all strain amplitudes, indicating cyclic softening behavior [10,11,23]. Taking the TMF cycles of alloy 319 at 0.4% strain amplitude as an example (Figure 2e), the plastic strain increased from 0.36% after the second cycle to 0.45 and 0.51% at the mid-life and final cycles, respectively, while the maximum tensile stress decreased from 236 MPa after the second cycle to 198 and 156 MPa at the mid-life and final cycles, respectively.

On the other hand, differences between the two experimental alloys were also observed. Figure 2 shows that the 319 alloy always exhibited higher maximum/minimum stress than the 356 alloy, whereas the extent of the strain range at zero stress (plastic strain) was always greater for the 356 alloy than for the 319 alloy. For example, the maximum and minimum stresses of the 319 alloy during the mid-life cycle under a 0.6% strain amplitude (Figure 2g,h) were 206 and 121 MPa, respectively, which were significantly higher than the respective values of 96 and 86 MPa obtained for the 356 alloy. In contrast, the plastic strain in the 356 alloy was 0.8%, higher than the value of 0.6% in the 319 alloy. These differences can be attributed to the different mechanical properties of the two alloys, with the 319 alloy with a high Cu content exhibiting higher strength but lower elongation than the 356 alloy [5,24,25,26].

**Figure 3:**
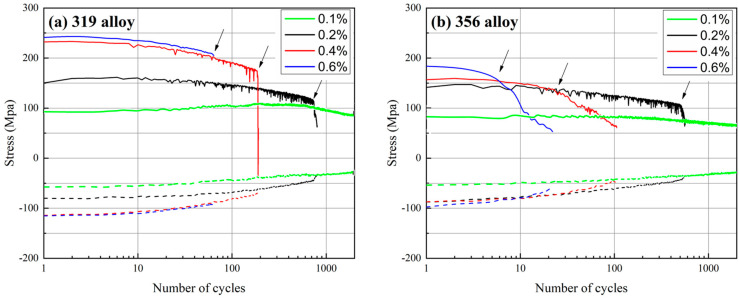
Evolution of the maximum tensile and compression stresses as a function of the cycle number at different strain amplitudes: (**a**) 319 alloy; (**b**) 356 alloy.

Figure 3 shows the evolution of the maximum tensile and compression stresses of the 319 and 356 alloys as a function of the fatigue cycles at different strain amplitudes. The maximum tensile stress for both alloys was always higher than the compression stress, which was due to the higher strength at lower temperatures in OP-TMF [11,23]. Additionally, the maximum tensile/compression stress generally decreased with increasing numbers of cycles, indicating cyclic softening for both alloys, which is also reflected in Figure 2.

In addition to the common phenomena in the two alloys, Figure 3 also shows some differences between the two. First, similarly to Figure 2, the maximum tensile/compression stresses of the 319 alloy were always higher than those of the 356 alloy. For example, the initial maximum tensile and compression stresses of the 319 alloy at a strain amplitude of 0.6% were 245 and 110 MPa, respectively, which were higher than the respective values of 190 and 97 MPa obtained for the 356 alloy. Second, the decrease in stress as a function of the number of cycles differed for the two alloys. As indicated by the arrows in Figure 3, the point where the softening rate exhibited a sharp change was defined as the inflection point at which the TMF could generally be divided into two different stages. Stage I spanned from the initial fatigue cycle to the inflection point. In this stage, the stress level initially increased during the first few cycles and then gradually decreased with a low softening rate. Stage II spanned from the inflection point to the point where the specimen failed, at which point the softening rate significantly increased. In this stage, the stress sharply decreased with increasing numbers of TMF cycles, which can be attributed to the occurrence of large cracks that exceeded the critical crack length [11]. The 356 alloy always exhibited an earlier inflection point than the 319 alloy, especially under higher strain amplitudes (0.4 and 0.6%), which resulted in early fatigue damage in the 356 alloy and, hence, reduced total fatigue life. For an improved comparison, the proportions of stage I in the total lifetimes of the two alloys were calculated, and the results are listed in Table 3. The table shows that stage I accounted for more than 90% of the total lifetime of the 319 alloy at all the tested strain amplitudes, even reaching 100% at the strain amplitude of 0.6%, which may indicate that there was little macro-damage before the end of the TMF test. However, the proportion of stage I in the 356 alloy was considerably lower, especially at higher strain amplitudes. As shown in Table 3, stage I accounted for only 22 and 27% of the total lifetime of the 356 alloy at the 0.4 and 0.6% strain amplitudes, respectively, indicating that the 356 alloy may have been subjected to earlier severe macro-damage during TMF cycling.

**Figure 4:**
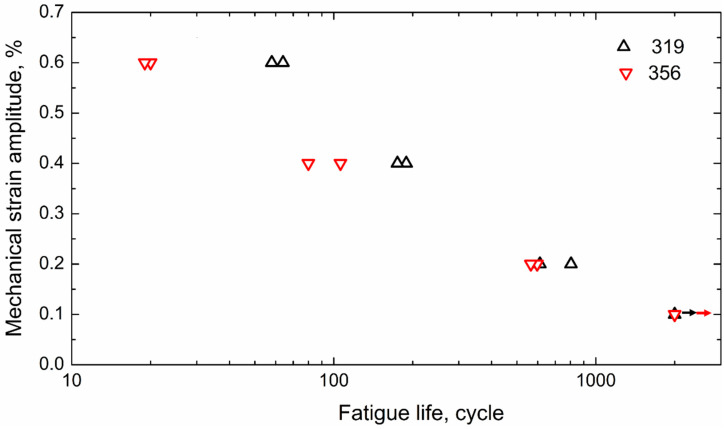
OP-TMF lifetimes of two experimental alloys under various strain amplitudes.

Figure 4 summarizes the OP-TMF lifetimes of the 319 and 356 alloys under different strain amplitudes. Generally, the fatigue life decreased with increasing strain amplitude, and both alloys exhibited approximately linear relations when plotted on a log–log scale. Both alloys reached the limit of 2000 cycles at a strain amplitude of 0.1%; however, the 319 alloy exhibited increased fatigue life starting at the 0.2% strain amplitude, especially at higher strain amplitudes (0.4 and 0.6%). As shown in Figure 4, the average fatigue life of the 319 alloy at a 0.2% strain amplitude was 700 cycles, which was slightly higher than the 580 cycles obtained for the 356 alloy. The 319 alloy exhibited a significantly longer average fatigue life than the 356 alloy at higher strain amplitudes, demonstrating two- and threefold longer average TMF lifetimes at the 0.4 (183 versus 90) and 0.6% strain amplitudes (62 versus 20), respectively. Therefore, the TMF resistance of the 319 alloy was generally higher than that of the 356 alloy, especially when exposed to higher strain amplitudes.

### 3.2. Fracture Analysis

**Figure 5:**
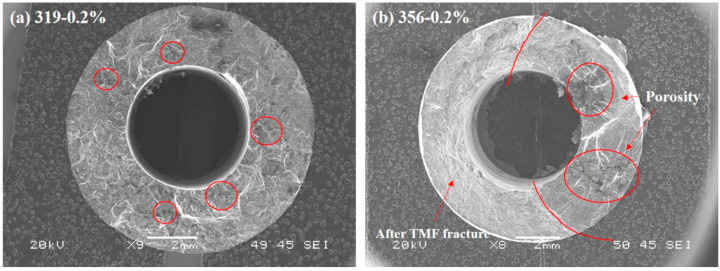
Fracture surface after 0.2% TMF test: (**a**) 319 alloy; (**b**) 356 alloy.

Figure 5 shows overall views of the fracture surfaces of the 319 and 356 alloys after TMF cycling at 0.2% strain amplitude. Porosity was observed on both fracture surfaces, as marked by the red circles in Figure 5. The porosity in the 319 alloy was relatively low and evenly spread across the sample. Crack ridges were found on the fracture surface of the 319 alloy, indicating that the cracks may have nucleated at multiple initiation sites and merged together during fatigue failure. Large and continuous pores were observed on the fracture surface of the 356 alloy, and these could have served as crack nucleation sites and provided easier propagation paths for the crack [11,15,16,17], which was one of the likely reasons for the earlier inflection point in the 356 alloy (see Figure 3). It is worth noting that the left part in Figure 5b (356 alloy, marked by two red lines) was not the original TMF fracture surface but the tensile fracture surface from the pulling process after the TMF test, as the sample was not fully fractured when the TMF was terminated. As explained in the Materials and Methods section, the sample that remained unfractured upon termination of the test was pulled to full fracture.

**Figure 6:**
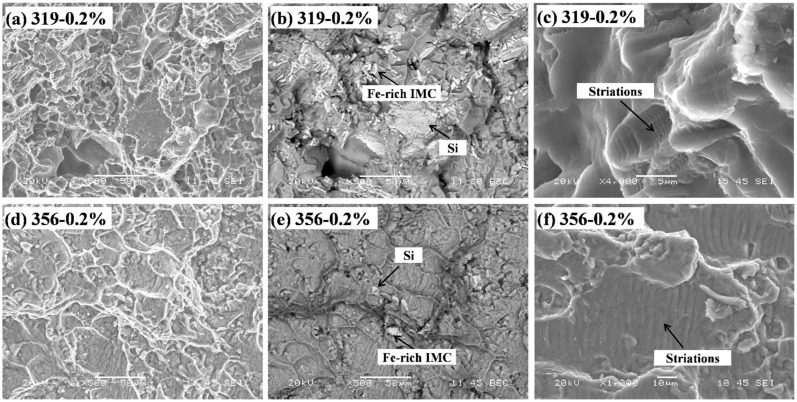
Fractography of fractured specimens after 0.2% TMF test: (**a**–**c**) 319 alloy; (**d**,**e**) 356 alloy; (**a**,**c**,**d**,**f**) SEM secondary electron images; (**b**,**e**) SEM backscatter images.

**Figure 7:**
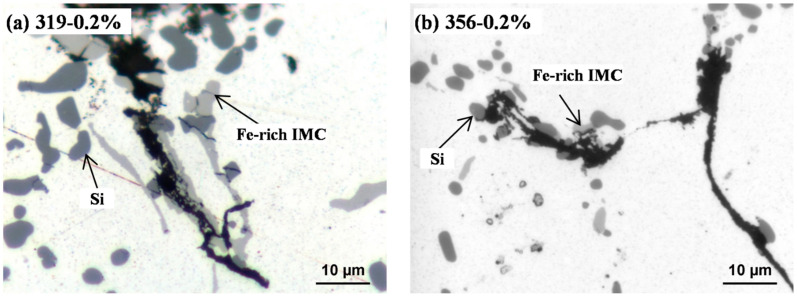
Optical cross-section images of the fracture surfaces showing the cracks in the (**a**) 319 alloy and (**b**) 356 alloy after the 0.2% TMF test.

Figure 6 shows the fracture faces at the crack propagation areas in the 319 and 356 alloys at 0.2% strain amplitude. As shown in Figure 6a,d, both fracture faces appeared as ductile fractures with dimples in the matrix. However, the dimples in the 319 alloy were smaller than those in the 356 alloy, indicating its lower plastic strain. The SEM backscatter images in Figure 6b,e show that a number of bright phases were found on the fracture surface, as marked by the arrows, which were identified as Fe-rich intermetallics and eutectic Si particles [24,25]. The 319 alloy (Figure 6b) exhibited more Fe-rich intermetallics than the 356 alloy (Figure 6e), which was attributed to its higher Fe content (0.3 and 0.1% in the 319 and 356 alloys, respectively (Table 1)). As reported in [5,11,17], these hard intermetallic phases could serve as nucleation sites for cracks as a result of their detachment from the aluminum matrix or by rupturing themselves. This was confirmed by the observation of the cross-sectional fracture surface in Figure 7, in which several broken eutectic Si particles and Fe-rich intermetallic phases were observed in the 319 and 356 alloys after the TMF tests. Table 4 summarizes the characteristics of the Fe-rich intermetallics and eutectic Si particles in both alloys in the T7 condition (before TMF). The eutectic Si in the 356 alloy was more globular and had a higher area fraction, whereas the area fraction of the Fe-rich intermetallics in the 319 alloy was double that in the 356 alloy. However, the total area fractions of the eutectic Si and Fe-rich intermetallics in both alloys were similar, indicating their similar contributions to the nucleation and propagation of cracks during TMF deformation. Additionally, striations were observed in both the 319 (Figure 6c) and 356 (Figure 6e) alloys. The interspacing of the striations in the 356 alloy was larger than that in the 319 alloy, indicating faster fatigue propagation during TMF cycling [27].

## 4. Discussion

### 4.1. Microstructural Evolution during TMF

**Figure 8:**
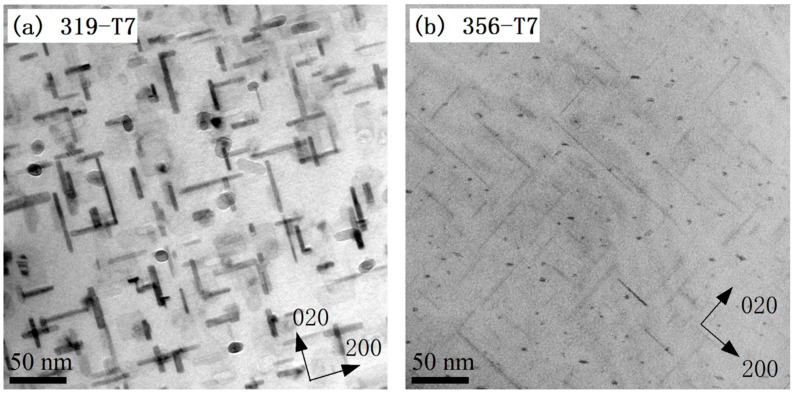
Bright-field TEM images showing precipitate microstructure after T7 (before TMF) for (**a**) θ′-Al_2_Cu in the 319 alloy and (**b**) β′-MgSi in the 356 alloy.

As shown in Figure 3, the maximum tensile and compression stresses for both alloys gradually decreased with the increasing numbers of cycles at all strain amplitudes. This cyclic softening behavior during TMF was likely related to the evolution of the precipitates during the TMF cycling [12,16,18,20,23]. As typical precipitation-strengthening alloys, the precipitates in both alloys play an important role in their mechanical properties. Figure 8 shows the initial precipitate microstructures of the two alloys in the T7 condition (before TMF). A large number of nano-sized precipitates were visible in the aluminum matrixes of both alloys, which were identified as mainly the plate-like θ′-Al_2_Cu phase in the 319 alloy and the needle-like β′-MgSi phase in the 356 alloy, consistent with reports from the literature [5,16,25].

**Figure 9:**
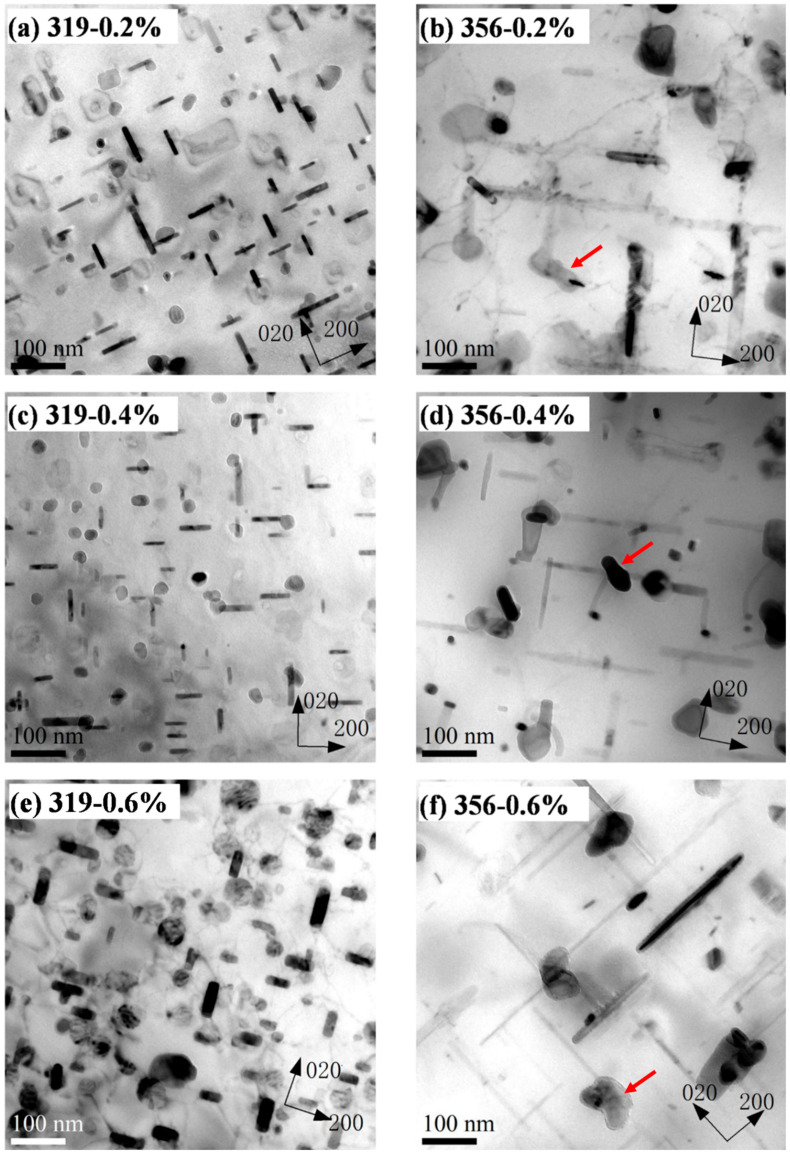
Bright-field TEM images showing precipitate coarsening after TMF at various strain amplitudes: (**a**,**c**,**e**) 319 alloy and (**b**,**d**,**f**) 356 alloy.

Figure 9 presents the precipitate microstructures of the two alloys after TMF cycling at various strain amplitudes. In general, all the precipitates in the two alloys coarsened during TMF cycling. Figure 9 shows that, after TMF cycling, the plate-like θ′-Al_2_Cu in the 319 alloy became rod-like θ′-Al_2_Cu, whereas the needle-like β′-MgSi coarsened to rod-like β′-MgSi. However, their number densities decreased significantly as their size increased. As shown in Table 5, the number density of the precipitates in the 319 alloy after TMF cycling at 0.2% strain amplitude decreased from 4451 to 501 μm^−1^ and the length increased from 48 to 115 nm compared to the T7 condition (before TMF). A similar phenomenon was also observed in the 356 alloy. Though both the θ′-Al_2_Cu and β′-MgSi coarsened during the TMF, different coarsening behaviors were observed for the two alloys. Compared to the evolution of θ′-Al_2_Cu in the 319 alloy shown in Figure 9, the size and number-density changes for the β′-MgSi during TMF were significantly larger, especially at the higher strain amplitude (0.6%) (compare Figure 9e,f). As shown in Table 5, after TMF cycling at the 0.6% strain amplitude, the length of the θ′-Al_2_Cu in the 319 alloy increased from 48 to 80 nm and the number density decreased from 4451 to 691 μm^−1^. On the other hand, the size of the β′-MgSi in the 356 alloy increased from 70 to 205 nm and the number density significantly decreased from 19,144 to 394 μm^−1^. Additionally, a small part of the β′-MgSi in the 356 alloy transformed into equilibrium β-Mg_2_Si after TMF cycling, as indicated by the red arrows in Figure 9b,d,f. The rod-like θ′-Al_2_Cu remained the predominant precipitate in the 319 alloy after TMF cycling, which indicated that the precipitates in the 356 alloy exhibited a higher coarsening rate than those in the 319 alloy.

The coarsening of precipitates during TMF cycling was mainly related to their TMF behavior. In this study, the coarsening rates of both the precipitates were evaluated using the classical Lifshitz–Slyozov–Wagner model, which is expressed by Equation (1) [28]:

(1) $$L^{n}-L_{0}^{n}=k\cdot t$$

where *L* is the average half-length of the precipitates after TMF cycling, *L*_0_ is the average half-length of the precipitates in the T7 condition (before TMF), *k* is the coarsening rate constant, *t* is the time, and *n* is the temporal exponent. It has been reported [29,30] that the growth of θ′-Al_2_Cu and β′-MgSi precipitates is prone to obeying the *t*^1/2^ law (*n* = 2) under the dominant coarsening of the interface reaction. Thus, *n* = 2 was adopted in this study. Additionally, the value of *t* is normally taken as the time elapsed at constant temperature. However, in this study, the temperature in each fatigue cycle changed, meaning that the coarsening rate per second could not be applied here due to the temperature variation between 60 and 300 °C in each cycle. For simplification, the value of *t* was modified to the number of TMF cycles in this study, and the value of *k* in Equation (1) was calculated as the coarsening rate for the precipitates per cycle. The *k* values of the precipitates in the two alloys were calculated using the data in Table 5, and the results are summarized in Table 6.

As shown in Table 6, *k* generally increased with an increasing strain amplitude, indicating that a higher strain amplitude resulted in a higher precipitate coarsening rate. This was attributed to the heavy dislocations in the matrix, which were generated under the high-strain-amplitude conditions, accelerating the coarsening of the precipitates [31,32]. However, the 319 alloy exhibited a significantly lower coarsening rate than the 356 alloy at each strain amplitude, and the differences in *k* between the two alloys also increased with the strain amplitude. For example, *k* in the 319 alloy at a 0.2% strain amplitude was 3.45, which was only one tenth of the value of 43.85 obtained for the 356 alloy. It was even found that *k* in the 319 alloy at the 0.4 and 0.6% strain amplitudes decreased to levels 1/5th (6.2 versus 87.3) and 1/30th (16 versus 463), respectively, of those in the 356 alloy.

The lower coarsening rate for θ′-Al_2_Cu in the 319 alloy resulted in a slower decrease in the maximum stress during TMF (Figure 3), which has also been reported in the literature [11,18]. The lower coarsening of the precipitates and slower decrease in the maximum stress could stabilize both the matrix and stress level and, hence, enhance the TMF endurance of the 319 alloy (Figure 4). The coarsening rate of the precipitates per cycle in the 319 alloy, especially at the high strain amplitude (0.6%), was significantly lower than that in the 356 alloy, leading to a longer TMF life for the 319 alloy. In contrast, the plastic strain in the 356 alloy during TMF cycling was higher than that in the 319 alloy, and the rate of decrease in the maximum stress was also higher (Figure 3), which can result in a greater portion of the sample deforming during TMF loading, as well as the buckling effect in the gauge area, which further produces early failures [11]. This phenomenon occurred more frequently at the higher strain amplitudes. As shown in Figure 3, the stress inflection point of the 356 alloy at the strain amplitude of 0.6% occurred after only a few cycles, resulting in a significantly shorter TMF life relative to that of the 319 alloy.

### 4.2. TMF Lifetime Prediction

**Figure 10:**
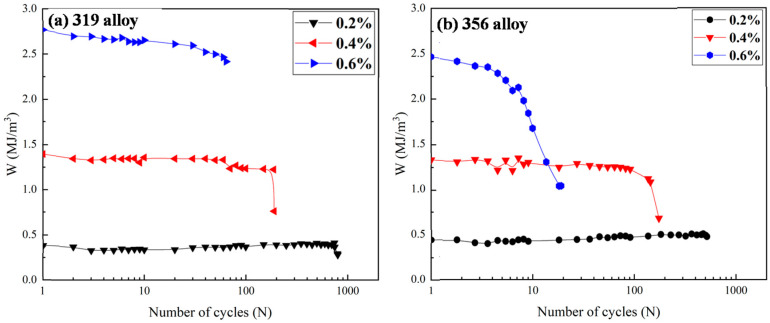
Evolution of hysteresis loop area (*W*) as a function of cycles for (**a**) 319 alloy and (**b**) 356 alloy.

Thermo-mechanical fatigue lifetimes are difficult to predict due to the complex deformation under both strain and temperature cycling during TMF. Various models have been developed based on different mechanisms, including the Neu–Sehitoglu model based on creep, fatigue, and oxidation damage [33]; the Miller model based on the accumulation of the damage rate [34]; the J-integral model based on fracture mechanics [35]; and the energy-based model based on the dissipated energy per cycle [8,36,37,38]. Among these models, the energy-based model is widely accepted as a more suitable approach to predict TMF lifetimes [8,36,37,38,39,40]. The hysteresis energy in the energy-based model involves both the strain and stress amplitudes and can be approximated as the product of the plastic strain range (Δ*ε_p_*) and the stress range (Δ*σ*) using Equation (2) [8,40]:

(2) $$W_{i}=\int \sigma d\varepsilon \approx \Delta \varepsilon_{p}\cdot \Delta \sigma$$

where *W_i_* represents the hysteresis energy variable of the *i*th cycle. The evolution of the hysteresis energy (*W*, calculated using the hysteresis loop area) with increasing numbers of cycles under various strain amplitudes in the two alloys, as determined using Equation (2), is shown in Figure 10. The hysteresis energy for both alloys involved both the strain and stress amplitudes and exhibited cyclic stability after a short period of adaptation for most of the strain amplitudes applied, confirming the greater rationality of the energy-based model. However, an early energy loss occurred in the 356 alloy after the first few cycles at the 0.6% strain amplitude, which may have been related to early crack formation during TMF loading.

On the other hand, the hysteresis energy (equal to the plastic strain energy) is also related to the fatigue life (*N_f_*), as shown in Equation (3) [8,36]:

(3) $$W_{s}=W_{0}\cdot N_{f}^{-1/\beta }$$

where *W_s_* is the saturation hysteresis plastic strain energy. As shown in Figure 10, the plastic strain energy reached the saturation stage after only a few cycles, which thus allowed for *W_s_* to be determined from the fatigue data after that point, and it was generally obtained at the mid-life cycle of the completed TMF tests. *W*_0_ and *β* are material parameters representing the fatigue damage capacity and fatigue damage exponent, respectively. They can be calculated from the log*W_s_* − log*N_f_* relationship [36,39]. In this way, the *W*_0_ and *β* calculated from the known fatigue life and plastic strain energy under a selection of strain amplitudes can be used with Equation (3) to estimate the TMF life under other strain amplitudes after only implementing a few cycles, such as 10; thus, the saturation plastic strain energy can be obtained, which is more time-efficient than running a full TMF test at each strain amplitude.

Due to the limited data in this study, the TMF behaviors at the 0.2 and 0.6% strain amplitudes were used to calculate *W*_0_ and *β*. The TMF life at the 0.4% strain amplitude was then estimated using Equation (3) and compared with the experimental value to validate the model. The data for both alloys after 2000 cycles at the 0.1% strain amplitude were obtained by triggering the settled limit of the test and could not be used to determine the *W*_0_ and *β*. Table 7 lists the calculated *W*_0_ and *β* values, which are similar to the reported values for Al-Si cast alloys [36]. The 319 alloy exhibited a higher fatigue damage capacity value (*W*_0_) than the 356 alloy. The exponent value (*β*) represents the relation between the TMF lifetime and hysteresis energy, and the higher *β* value for the 356 alloy indicates that the TMF lifetime decreased significantly with increasing hysteresis energy.

**Figure 11:**
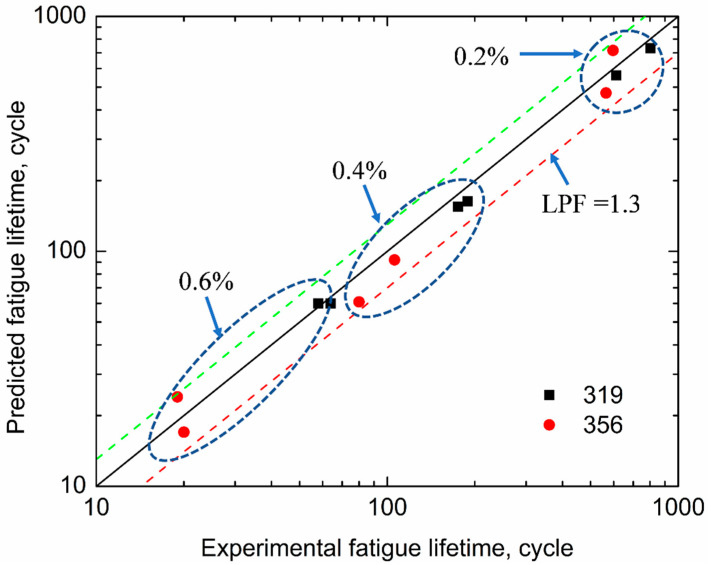
Comparison of predicted and experimental fatigue lifetimes for 356 and 319 alloys.

The predicted fatigue lifetime at the 0.4% strain amplitude was calculated using Equations (2) and (3), as well as the data in Table 7, and compared with the experimental fatigue life, as listed in Table 8. The results illustrate that the predicted lifetime corresponded well with the experimentally measured lifetime. Figure 11 presents the experimental and predicted TMF lifetimes at 0.2–0.6% strain amplitudes. All the predicted TMF lifetimes correlated with the low life prediction factor (LPF; approximately 1.3) obtained from the experimental life [36], confirming that the energy-based model was suitable for the prediction of the TMF lifetimes of the two alloys and had acceptable accuracy. Using the parameters from Table 7 and the TMF behavior shown in Figure 3, the TMF lifetimes of the 319 and 356 alloys at the strain amplitude of 0.1% were estimated to be 4152 and 4730 cycles, respectively, both of which were significantly higher than 2000 cycles.

## 5. Conclusions

This study investigated and compared the OP-TMF behaviors of two typical Al-Si cast alloys (319 and 356) and the following conclusions were drawn:

1. During TMF loading, asymmetrical hysteresis loops were observed in both alloys and the tensile portion was higher than the compression portion due to out-of-phase cycling;
2. Cyclic softening behavior was observed for the maximum stress in both alloys, but the rate of decrease in the cyclic stress in the 356 alloy was greater than that in the 319 alloy, especially at higher strain amplitudes. Moreover, the 356 alloy presented an earlier inflection point in the cyclic evolution of the cyclic stress;
3. The TMF resistance decreased with increasing strain amplitudes. The 319 alloy exhibited a longer TMF lifetime than the 356 alloy, especially at higher strain amplitudes;
4. The precipitates in both alloys coarsened during TMF cycling. The coarsening rate of the β′-MgSi in the 356 alloy per cycle was higher than that of the θ′-Al_2_Cu in the 319 alloy and increased at higher strain amplitudes;
5. The fatigue lifetime predicted using the energy-based model corresponded well with the experimental results, exhibiting a low life prediction factor of 1.3. However, the material parameters varied with the alloys, and the 356 alloy exhibited a lower fatigue damage capacity (*W*_0_) and a higher fatigue damage exponent (*β*).

## Figures and Tables

**Table 1:** Chemical compositions of the alloys studied (wt.%).

Alloy	Si	Cu	Mg	Mn	Fe	Ti	Sr	Al
319	5.93	3.34	0.12	0.284	0.307	0.11	0.0106	Bal.
356	7.27	0.60	0.34	0.206	0.109	0.21	0.0113	Bal.

**Table 2:** Heat treatment parameters for the alloys.

Alloy	Solution Treatment	T7 Aging Treatment
319	495 °C/4 h + 515 °C/2 h	200 °C/5 h
356	500 °C/4 h + 540 °C/2 h	

**Table 3:** Proportion of stage I in total TMF lifetimes of experimental alloys.

Strain Amplitude	319 Alloy	356 Alloy
0.2%	90%	85%
0.4%	96%	22%
0.6%	100%	27%

**Table 4:** Microstructure characteristics of experimental alloys under T7 condition (before TMF).

	319 Alloy	356 Alloy
Morphology of eutectic Si	Lamellar	Globular
Diameter of eutectic Si	4.0 μm	3.5 μm
Area fraction of eutectic Si	6.6%	8.8%
Area fraction of intermetallics (total)	1.6%	0.8%

**Table 5:** Characteristics of precipitates of the alloys before and after TMF.

Alloys	Conditions	Length (nm)	Width (nm)	Number Density (μm^−1^)
319 (θ′-Al_2_Cu)	T7 before TMF	48.1	4.3	4451.2
	After 0.2%	115.9	12.1	501.4
	After 0.4%	83.9	10.0	789.8
	After 0.6%	80.1	18.1	690.6
356 (β′-MgSi)	T7 before TMF	70.1	2.8	19144.8
	After 0.2%	331.1	14.9	191.1
	After 0.4%	204.7	14.6	253.7
	After 0.6%	204.8	12.6	394.0

**Table 6:** Coarsening rate during TMF under various strain amplitudes.

Strain Amplitude	*k* Value	
	319 Alloy	356 Alloy
0.2%	3.45	43.85
0.4%	6.22	87.32
0.6%	16.05	463.01

**Table 7:** Material parameters calculated with energy-based model.

	*W*_0_	*β*
319 alloy	107.7	1.09
356 alloy	5.27	2.28

**Table 8:** Experimental and predicted TMF lifetimes under 0.4% strain amplitude for the two alloys.

	319		356	
	Test 1	Test 2	Test 1	Test 2
Experimental	189	175	80	106
Predicted	163	155	61	92

## Data Availability

Supporting data are available upon reasonable request.
